# The *OsNAC25* Transcription Factor Enhances Drought Tolerance in Rice

**DOI:** 10.3390/ijms26104954

**Published:** 2025-05-21

**Authors:** Aohuan Yang, Qiong Luo, Lei Liu, Meihe Jiang, Fankai Zhao, Yingjiang Li, Bohan Liu

**Affiliations:** 1 College of Agronomy, Hunan Agricultural University, Changsha 410128, China; 2022-2025.yah@stu.hunau.edu.cn (A.Y.); 2871726181@stu.hunau.edu.cn (Q.L.); 1982954553@stu.hunau.edu.cn (L.L.); 13142358081@163.com (M.J.); 17789369834@163.com (F.Z.); 13755161912@163.com (Y.L.); 2 Yuelushan Laboratory, Changsha 410128, China

**Keywords:** rice, *OsNAC25*, abiotic stress, drought tolerance, transcriptome analysis

## Abstract

Drought represents a prevalent abiotic stress in terrestrial plants, frequently impairing crop growth and yield. In this paper, we characterized the functional role of *OsNAC25*, a member of the NAC transcription factor family, in drought tolerance. *OsNAC25* was predominantly localized in both the cytoplasm and nucleus, with its expression being markedly induced under drought conditions. Under severe drought stress, the overexpression of *OsNAC25* rice exhibited decreased malondialdehyde (MDA) levels, attenuated oxidative damage, and improved survival rate during the vegetative growth stage. The transcriptome analysis revealed that *OsNAC25* coordinates drought response through key pathways associated with phenylpropanoid biosynthesis, plant hormone signal transduction, and diterpenoid biosynthesis. Collectively, our findings highlight *OsNAC25* as a pivotal transcriptional regulator governing drought resistance in rice. This study not only provides a candidate gene for improving drought tolerance in rice but also offers valuable insights into the molecular mechanisms underlying drought adaptation in cereal crops.

## 1. Introduction

Rice (*Oryza sativa*), serving as the staple food for more than half of the global population, plays a pivotal role in ensuring food security through yield stability. Although substantial efforts have been made and notable progress achieved in enhancing rice productivity, agricultural production remains severely threatened by adverse climatic conditions, with drought being the most prominent stressor [1,2,3]. Climate change has led to increased frequency and intensity of drought events, posing significant challenges to rice cultivation. As a semi-aquatic crop particularly sensitive to water deficit, rice exhibits high vulnerability to drought stress, which has emerged as one of the predominant abiotic constraints limiting global rice production. The plant’s response to drought involves a complex regulatory network encompassing multifaceted physiological adaptations, biochemical adjustments, and molecular reprogramming [4,5,6]. Therefore, elucidating the mechanistic basis of drought response and adaptive strategies in rice plants holds great promise for developing high drought-resistant cultivars, which is crucial for sustainable agricultural production and global food security under changing climate scenarios.

NAC (NAM, ATAF1/2, and CUC2) transcription factors (TFs) have emerged as central regulatory players in plant stress responses [7]. Characterized by a conserved N-terminal DNA-binding domain and a highly divergent C-terminal transcriptional activation domain, this protein family exhibits distinctive structural features [8]. Studies have demonstrated the pivotal roles of NAC TFs in developmental regulation, while accumulating evidence confirms their integral involvement in plant responses to abiotic stresses [9]. In the context of drought resistance mechanisms in rice (*Oryza sativa*), NAC TFs orchestrate a multifaceted regulatory network [10,11,12]. Emerging evidence reveals distinct molecular strategies employed by specific NAC members to enhance drought tolerance: SNAC1 modulates stomatal movement to reduce water loss; *OsNAC066* activates antioxidant systems to alleviate oxidative damage; *OsXND1* and *OsNAC17* improve hydraulic conductivity through xylem development regulation and lignin biosynthesis, respectively; while *OsNAC15* coordinates stress signaling via ABA pathway mediation [13,14,15,16,17]. Notably, the drought-responsive functions of *OsNAC67*, *OsNAC45*, *OsNAC006*, *OsNAC14*, *OsNAC9*5, and *OsNAC22* have been systematically documented, collectively establishing this TF family as a master regulator in plant stress adaptation [16,18,19,20,21,22,23].

As a key member of the NAC transcription factor family, *OsNAC25* has been reported to regulate potassium deficiency responses and grain development. Furthermore, studies have demonstrated its role in coordinating starch biosynthesis homeostasis through physical interactions with *NAC20/26* proteins [24,25]. However, its potential involvement in drought resistance remained unexplored. In this investigation, the functional characterization of knockout and overexpression lines revealed that *OsNAC25* exhibits drought-inducible expression patterns and confers enhanced drought tolerance in rice (*Oryza sativa*). Transgenic plants overexpressing *OsNAC25* demonstrated significantly improved survival rates under extreme drought conditions during both vegetative and reproductive stages. Transcriptomic profiling further identified enriched pathways associated with drought resistance mechanisms. Intriguingly, unlike most nuclear-localized NAC TFs, *OsNAC25* displays dual localization in both the cytoplasm and nucleus. This unique subcellular distribution suggests a potential novel post-translational regulatory mechanism operating under drought stress conditions.

## 2. Results

### 2.1. OsNAC25 Is a Drought-Induced Transcription Factor with High Expression

**Figure 1:**
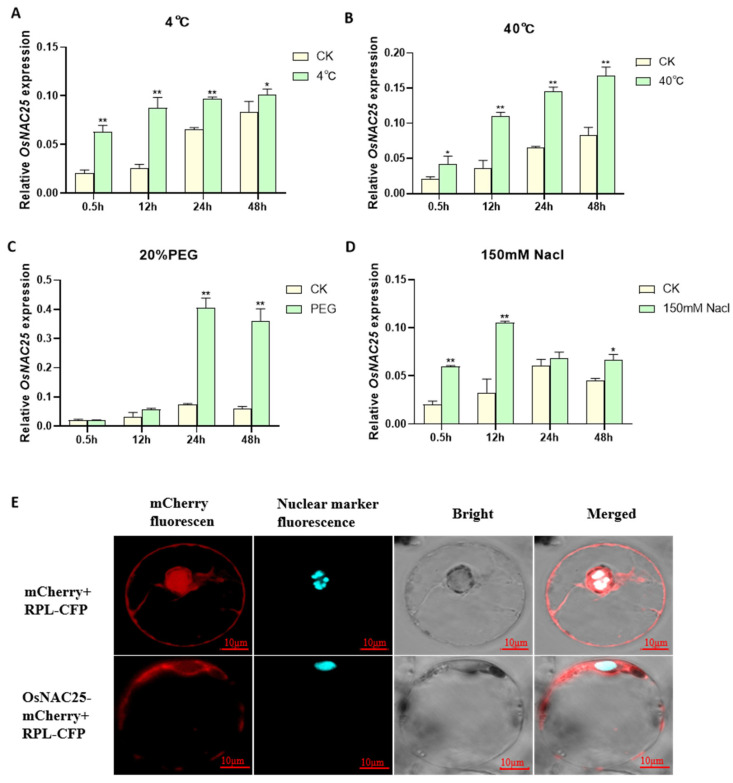
The expression of *OsNAC25* under abiotic stresses: (**A**) 4 °C, (**B**) 40 °C, (**C**) 20% PEG, and (**D**) 150 mM NaCl. Data show the mean ± SD of three replicates. Asterisks indicate significant differences between abiotic stresses and wild-type rice ZH11 using *t*-test (* *p* < 0.05, ** *p* < 0.01). (**E**) Subcellular localization of OsNAC25-mCherry +RPL-CFP in rice protoplasts. mCherry +RPL-CFP was used as a control.

To elucidate the spatiotemporal expression dynamics of *OsNAC25* under abiotic stress, we systematically analyzed its transcriptional responses in rice seedlings exposed to low temperature (4 °C), high temperature (40 °C), salt stress (150 mM NaCl), and simulated drought (20% PEG-6000) over a 48-hour period using qRT-PCR. The results demonstrate distinct temporal and spatial heterogeneity in *OsNAC25* regulation across different stress conditions (Figure 1). During the initial 12 h, low temperature, high temperature, and salt stress rapidly induced a significant upregulation of *OsNAC25*, with expression levels increasing by 3.4-fold (4 °C), 3.1-fold (40 °C), and 3.2-fold (NaCl) compared to the control (CK) (Figure 1A,B,D). In contrast, the PEG treatment showed no significant induction during this early phase (Figure 1C). Beyond 12 h, the regulatory patterns diverged markedly: both low-temperature and salt stress exhibited diminishing effects, with the low-temperature group showing only a 1.2-fold difference by 48 h and the salt-treated group reverting to CK levels by 24 h. While high-temperature stress maintained a 2.0-fold elevation at 48 h, the induction magnitude decreased by 34% compared to earlier peaks. Strikingly, PEG-induced drought triggered a unique sustained response, with *OsNAC25* expression surging sharply to 5.4-fold at 24 h and further increasing to 5.6-fold by 48 h, without the decline observed under other stresses. Collectively, *OsNAC25* is responsive to multiple abiotic stresses but displays a delayed, high-amplitude, and persistent induction specifically under drought-like conditions, contrasting with the transient responses to temperature or salt stress. These findings strongly implicate *OsNAC25* as a drought-inducible transcription factor potentially central to rice drought adaptation mechanisms.

To clarify the subcellular localization of *OsNAC25*, we constructed an *OsNAC25*-mCherry fusion expression vector and performed co-localization analysis using a rice protoplast transient expression system. The co-transfection of *OsNAC25*-mCherry with the nuclear marker RPL-CFP, alongside a negative control (empty mCherry vector co-transfected with RPL-CFP), revealed distinct localization patterns. Confocal microscopy showed that RPL-CFP signals were predominantly localized to the nucleus, while *OsNAC25*-mCherry fluorescence was distributed in both the nucleus and cytoplasm, with significant co-localization observed between *OsNAC25*-mCherry and RPL-CFP nuclear signals (Figure 1E). These results demonstrate that *OsNAC25* is a nucleo-cytoplasmic transcription factor, suggesting its dual regulatory role: transcriptional regulation in the nucleus and potential involvement in cytoplasmic stress-responsive signaling pathways.

### 2.2. Overexpression of OsNAC25 Enhances Drought Tolerance During the Vegetative Growth Stage in Rice

Based on qRT-PCR results indicating that *OsNAC25* may regulate drought-responsive seedling development, we investigated its functional role in drought tolerance by subjecting two-leaf-one-heart stage seedlings of *OsNAC25* overexpression lines (*OsNAC25*-OE), CRISPR/Cas9-generated knockout mutants (*OsNAC25*-cr), and *ZH11*plants to severe drought stress treatment.

**Figure 2:**
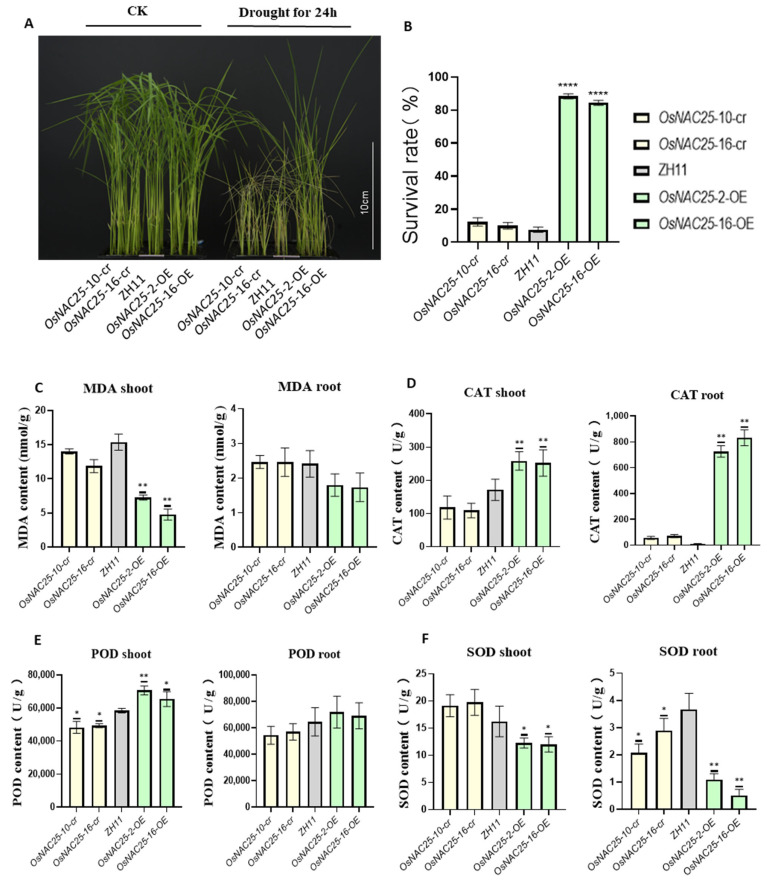
*OsNAC25* enhanced the tolerance of rice to drought stress. (**A**) *OsNAC25* transgenic rice plants were cultured in hydroponic boxes. The left side shows the control (CK), while the right side depicts the plants subjected to 24 h of drought stress after water withdrawal at the three-leaf-one-heart stage. (**B**) Phenotypic differences of *OsNAC25* under drought stress after two days; three biological replicates were conducted, and the error bars represent standard error (SE). Significance analysis was performed using a *t*-test (**** *p* < 0.0001). Antioxidant enzymatic activities under drought stress. (**C**) Malondialdehyde (MDA), (**D**) catalase (CAT), (**E**) peroxidase (POD), and (**F**) superoxide dismutase (SOD). Each sample was subjected to two biological replicates and four technical replicates, with the error bars representing standard error (SE). Significance analysis was performed using a *t*-test (* *p* < 0.05 or ** *p* < 0.01).

Consequently, the seedlings at the two-leaf-one-heart stage were subjected to severe drought stress by withholding water. Within 12 h, visible symptoms of wilting and chlorosis emerged in leaves, and by 24 h, pronounced phenotypic divergence became evident: nearly all *OsNAC25-cr* mutants and ZH11 plants exhibited severe wilting, whereas the *OsNAC25*-OE lines remained upright (Figure 2A). Following 24 h of drought treatment, the plants were re-watered and allowed to recover for one week. Survival rates were quantified, revealing that *OsNAC25-OE* lines displayed significantly higher survival (86%) compared to *OsNAC25-cr* (9.5%) and ZH11 (5.8%) plants (Figure 2B). In summary, the overexpression of *OsNAC25* substantially enhances drought tolerance in rice.

Antioxidant enzymes play a critical role in scavenging reactive oxygen species (ROS) and enhancing plant stress tolerance. To investigate the role of *OsNAC25* in mitigating oxidative stress under drought conditions, we analyzed the activities of key antioxidant enzymes—including catalase (CAT), peroxidase (POD), and superoxide dismutase (SOD)—as well as the malondialdehyde (MDA) content in the roots and leaves of ZH11, *OsNAC25* mutants (*OsNAC25-cr*), and *OsNAC25* overexpression lines (*OsNAC25-OE*).

Under drought stress, *OsNAC25-OE* lines exhibited significantly higher CAT and POD activities compared to the ZH11 and *OsNAC25-cr* mutants (Figure 2D,E). CAT catalyzes the decomposition of hydrogen peroxide (H_2_O_2_) into water and oxygen. In *OsNAC25-OE* plants, CAT activity increased by 1.5-fold in leaves and 778.1-fold in roots relative to ZH11. Similarly, POD, which scavenges peroxides, showed a 1.16-fold increase in leaf activity in the *OsNAC25-OE* lines compared to ZH11, though root POD activity showed no statistically significant enhancement. Notably, SOD levels in *OsNAC25-OE* lines were lower than those in the ZH11 and *OsNAC25-cr* mutants (Figure 2F), suggesting that *OsNAC25* may regulate antioxidant pathways through compensatory mechanisms or differential modulation. Despite reduced SOD levels, *OsNAC25-OE* lines displayed a significantly lower MDA content than the ZH11 and *OsNAC25-cr* plants, with leaf MDA levels reduced to 0.39-fold and root MDA to 0.73-fold of ZH11 values (Figure 2C), indicating attenuated oxidative damage overall.

Therefore, *OsNAC25* overexpression mitigates the toxic effects of reactive oxygen species (ROS) by enhancing antioxidant enzyme activities, thereby reducing oxidative damage in plant cells.

### 2.3. Overexpression of OsNAC25 Enhances Drought Tolerance During the Reproductive Growth Stage in Rice

**Figure 3:**
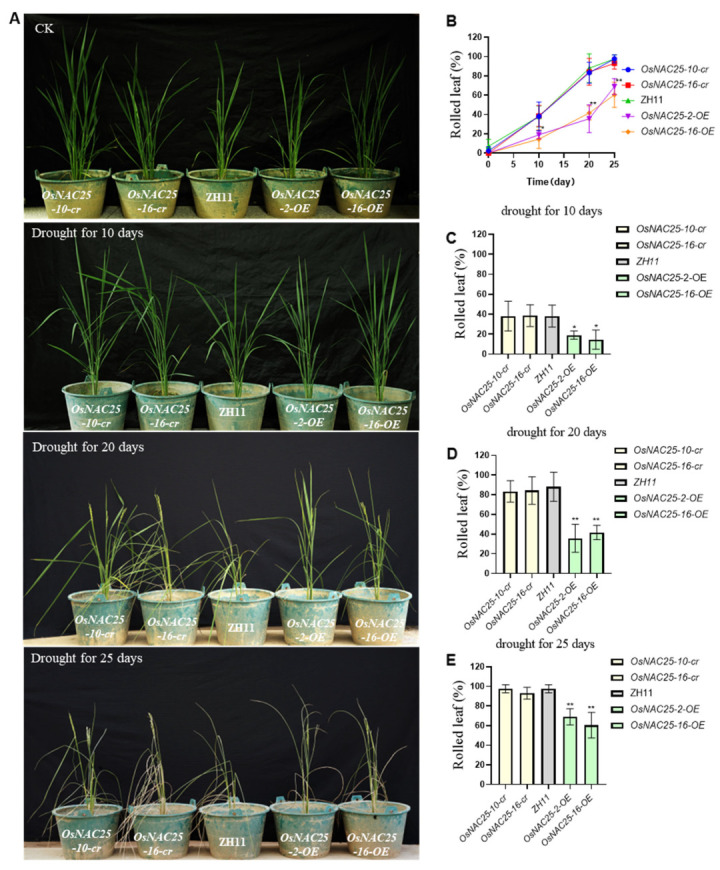
Overexpression of *OsNAC25* significantly enhances drought tolerance in rice. (**A**) Two *OsNAC25* knockout mutant lines (*OsNAC25*-cr), two overexpression lines (*OsNAC25*-OE), and ZH11 plants were subjected to complete drought stress starting at the booting stage. Phenotypic analyses were conducted at three time points: day 10, day 20, and day 25 of drought treatment. (**C**) By day 10 of drought, *OsNAC25*-OE lines exhibited a lower leaf rolling rate compared to *OsNAC25*-cr and ZH11 plants. (**D**,**E**) At days 20 and 25, leaf rolling rates in *OsNAC25*-OE lines were significantly reduced relative to the *OsNAC25*-cr and ZH11 plants. (**B**) A line graph illustrates the progressive decline in leaf turgidity across genotypes over the drought period, demonstrating the superior drought resilience of the *OsNAC25*-OE lines. All experiments included three independent biological replicates. Error bars represent standard error (SE) of the mean. Statistical significance was determined by a Student’s *t*-test (* *p* < 0.05, ** *p* < 0.01).

*OsNAC25* enhances drought tolerance during both the seedling and mature stages. To further investigate the role of *OsNAC25* in mature plants, we subjected *OsNAC25-OE*, *OsNAC25-cr*, and ZH11 plants to 25 days of drought stress starting at the post-booting stage and analyzed their phenotypes (Figure 3A). Leaf rolling rates were quantified, revealing that *OsNAC25-OE* lines exhibited a significantly lower rate (64.7%) compared to the *OsNAC25-cr* mutants (95.3%) and ZH11 (97.6%) (*p* < 0.05; three independent biological replicates). These results indicate that *OsNAC25-OE* plants retain turgid leaves for a markedly extended duration under prolonged drought stress. Collectively, our findings demonstrate that the *OsNAC25* transcription factor functions in both seedling and mature stages to significantly enhance drought tolerance in rice.

### 2.4. Gene-Editing Knockout and Overexpression of OsNAC25 Alter the Rice Transcriptome Profile

To elucidate the regulatory network of *OsNAC25* under drought stress, RNA sequencing (RNA-seq) was performed on aerial tissues of 10-day-old seedlings from *OsNAC25*-OE lines, *OsNAC25*-cr mutants, and ZH11 (wild type) under both drought and control conditions. Sequencing was conducted using the Qsep400 high-throughput platform, generating 110.48 Gb of clean data, with each sample yielding ≥5.75 Gb of high-quality reads (Q30 base percentage ≥93.07%). Clean reads were aligned to the reference genome, achieving mapping efficiencies ranging from 93.69% to 98.50% (Supplementary Table S1). Subsequent analyses included alternative splicing prediction, gene structure optimization, and novel gene discovery, identifying 3737 novel genes, of which 2483 were functionally annotated.

To validate RNA-seq results, eight differentially expressed genes (DEGs) were randomly selected from *OsNAC25-OE*, *OsNAC25-cr*, and ZH11 under drought and control conditions for qRT-PCR analysis. The expression levels of all eight genes were consistent with RNA-seq data (Supplementary Figure S1), confirming the reliability of our transcriptomic findings.

**Figure 4:**
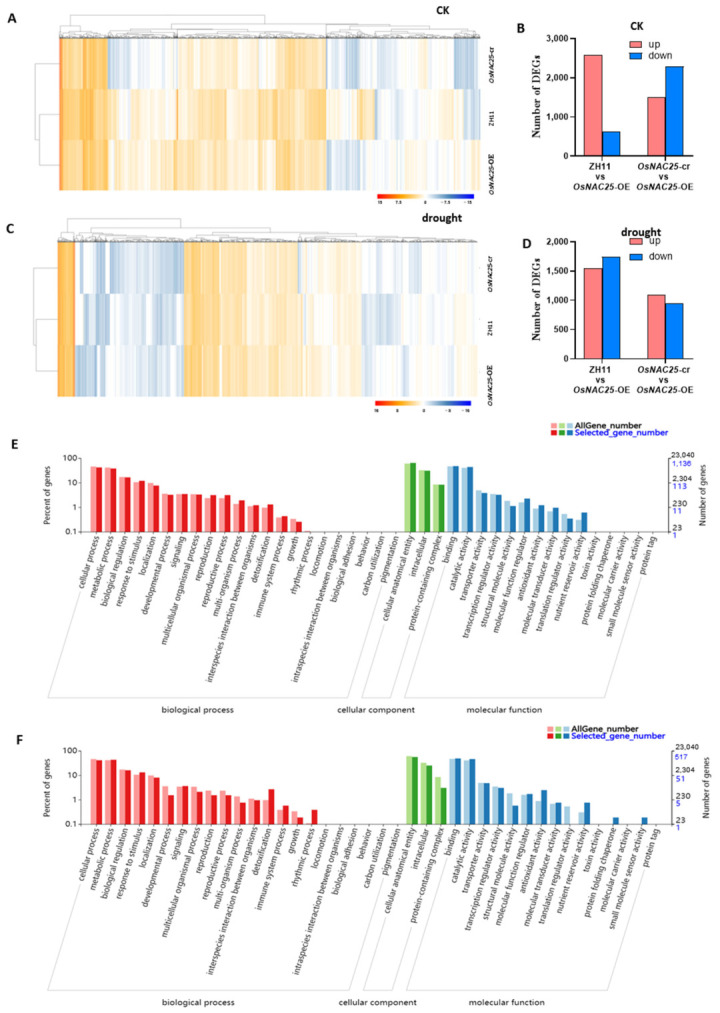
Genome-wide expression changes in *OsNAC25* overexpression and knockout rice lines. (**A**,**B**) Quantitative analysis of DEGs among ZH11, *OsNAC25*-cr, and *OsNAC25*-OE lines under control conditions, based on RNA-seq expression profiles. RNA was extracted from three independent biological replicates per genotype. (**C**) Hierarchical clustering of DEGs between ZH11, *OsNAC25*-cr, and *OsNAC25*-OE lines under drought stress. *OsNAC25* overexpression caused marked changes in gene expression compared to ZH11 and mutant lines. The color scale denotes log2(FPKM) values. (**D**) Statistical summary of DEGs among ZH11, *OsNAC25*-cr, and *OsNAC25*-OE lines under drought stress, derived from RNA-seq data. RNA was isolated from three independent replicates per genotype. (**E**) Gene ontology (GO) functional classification of shared DEGs between ZH11, *OsNAC25*-cr, and *OsNAC25*-OE lines under control conditions. The *x*-axis displays selected GO terms, and the *y*-axis indicates the percentage of annotated genes (number of genes in a term/total genes × 100%). (**F**) GO functional classification of shared DEGs under drought stress. The *x*-axis shows enriched GO terms, and the *y*-axis represents the proportion of genes assigned to each term.

The RNA-seq results demonstrate significant alterations in gene expression under both experimental conditions (Figure 4A,C). Under normal conditions, 2570 genes were upregulated and 615 genes downregulated in the *OsNAC25-OE* lines compared to the wild-type ZH11, while 1506 genes were upregulated and 2285 genes downregulated relative to *OsNAC25-cr* mutants (Figure 4B). Following drought stress, *OsNAC25-OE* exhibited 1548 upregulated and 1743 downregulated genes versus ZH11, and 1089 upregulated and 942 downregulated genes compared to the *OsNAC25-cr* lines (Figure 4D).

The Venn analysis identified 1601 constitutively differentially expressed genes (DEGs) shared between *OsNAC25*-overexpressing (*OsNAC25*-OE) transgenic lines and control groups (ZH11 wild-type + *OsNAC25*-CRISPR) under well-watered conditions (Supplemental Figure S2), indicating putative basal regulatory functions of *OsNAC25* before drought exposure. Post-drought comparative transcriptomics revealed 787 overlapping DEGs consistently regulated between *OsNAC25*-OE and control genotypes (Supplemental Figure S2).

The gene ontology (GO) enrichment analysis was performed on the 1601 (pre-drought) and 787 (post-drought) differentially expressed genes (DEGs) to identify associated biological processes (Figure 4E,F). DEGs related to biological processes were primarily associated with cellular processes, metabolic processes, biological regulation, and response to stimuli. Cellular component-associated DEGs were predominantly linked to cellular anatomical entities and intracellular organelle functions. Molecular function-enriched DEGs mainly involved binding and catalytic activity. Among these, cellular/metabolic processes, biological regulation, response to stimuli, cellular anatomy/intracellular components, binding, and catalytic activity exhibited the most significant differential expression (Figure 4E,F).

### 2.5. OsNAC25 Mediates Transcriptional Responses Under Drought Stress

The GO enrichment analysis identified 29 significantly enriched terms between *OsNAC25*-cr mutants and *OsNAC25*-OE lines. A further investigation of DEG biological functions via KEGG (Kyoto Encyclopedia of Genes and Genomes) pathway enrichment highlighted the most impacted pathways: phenylpropanoid biosynthesis, cyanogenic amino acid metabolism, taurine and hypotaurine metabolism, plant hormone signal transduction, brassinosteroid biosynthesis, peroxisome activity, circadian rhythm in plants, carbon metabolism, carbon fixation in photosynthetic organisms, diterpenoid biosynthesis, and starch and sucrose metabolism (Supplementary Figures S3–S6).

The KEGG pathway analysis identified eight prioritized pathways for further investigation, with the heatmap analysis focusing on key genes associated with cellular/metabolic processes, biological regulation, response to stimuli, cellular anatomy/intracellular components, binding, and catalytic activity. Among these, phenylpropanoid biosynthesis and taurine/hypotaurine metabolism emerged as the most significantly impacted pathways.

**Figure 5:**
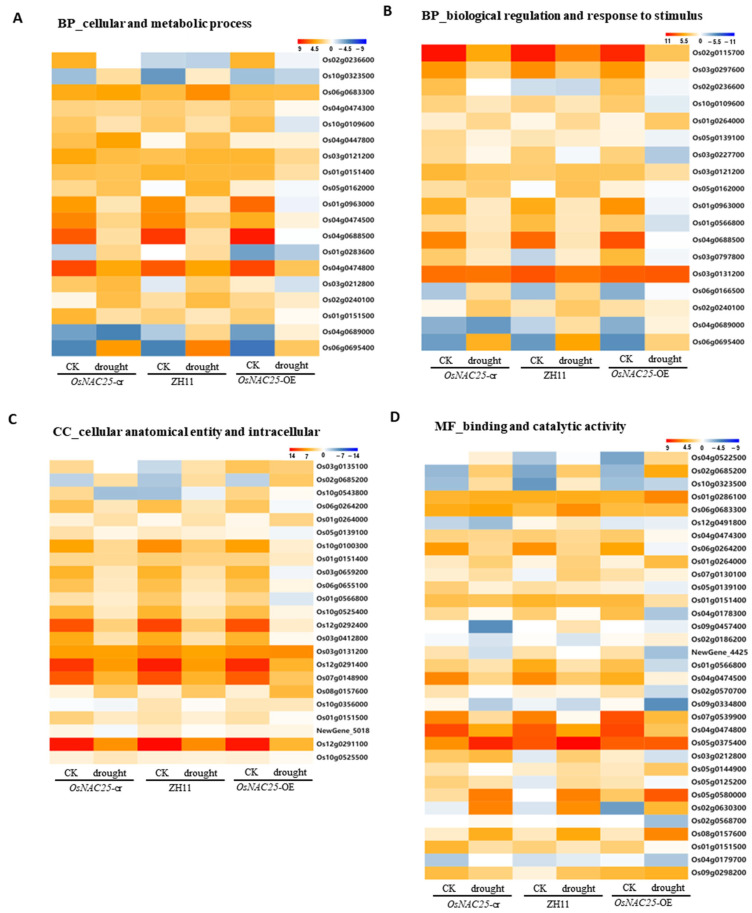
Transcriptional profiling of systemically regulated genes in ZH11, *OsNAC25-cr*, and *OsNAC25-OE* lines under drought stress. (**A**) Cellular and metabolic processes. (**B**) Biological regulation and response to stimuli. (**C**) Cellular anatomy and intracellular components. (**D**) Binding and catalytic activity. Heatmaps display log2 fold-change (FC) values of differentially expressed genes (DEGs) in *OsNAC25-cr* mutants, ZH11 (wild type), and *OsNAC25-OE* lines under control (CK) and drought-treated conditions.

Key genes in phenylpropanoid biosynthesis included peroxidase *OsPrx30* (*Os02g0240100*) and β-glucosidase *Os3BGlu6* (*Os03g0212800*), both linked to drought resistance, while glutamate decarboxylase *OsGAD2* (*Os04g0447800*) was central to taurine/hypotaurine metabolism (Figure 5A). Plant hormone signaling transduction pathways exhibited marked transcriptional changes, notably in auxin transcriptional repressor *OsIAA14* (*Os03g0797800*), PGL1 antagonist *OsPIL16* (*Os05g0139100*), and ABA receptor *OsPYL4* (*Os03g0297600*) (Figure 4B). Enhanced ROS scavenging capacity was corroborated by the differential expression of catalase *OsCatC* (*Os03g0131200*) in peroxisome pathways (Figure 4B). In the binding and catalytic activity pathways, diterpenoid biosynthesis gene *OsKSL12* (*Os02g0568700*) and pyruvate phosphate dikinase *OsPPDKA* (*Os03g0432100*)—a carbon metabolism gene implicated in drought regulation—showed significant expression shifts (Figure 5D). Intracellular pathways were further enriched for catalase *OsCatC* (*Os03g0131200*) and ferredoxin *OsFdC1* (*Os03g0659200*), the latter maintaining redox homeostasis via photosynthetic electron transport under drought-induced ROS accumulation (Figure 5D). The key genes identified in this study are systematically presented (Table 1), which documents their differential expression profiles (upregulated/downregulated) under drought stress between: (i) wild-type ZH11 vs. overexpression lines, and (ii) knockout mutants vs. overexpression lines.

## 3. Discussion

As one of the largest transcription factor families in plants, the rice NAC family comprises 151 members [10,26]. This study focused on the functional characterization of *OsNAC25*, revealing its dual expression activity in both the nucleus and cytoplasm, with its transcript levels being significantly induced by multiple abiotic stresses, particularly showing the most pronounced induction under drought stress. To elucidate its drought-responsive mechanism, we generated *OsNAC25* gene-edited materials (selecting two homozygous mutants, *OsNAC25*-10-cr and *OsNAC25*-16-cr) and overexpression lines (choosing those with highest expression levels, *OsNAC25*-2-OE and *OsNAC25*-16-OE) (Supplementary Figure S7A,B). The phenotypic analysis demonstrated that overexpression lines exhibited markedly enhanced drought tolerance compared to ZH11under water deficit, whereas no significant phenotypic divergence was observed between gene-edited materials and ZH11 (Figure 2A and Figure 3A). A further investigation revealed a substantially higher expression abundance of *OsNAC25* in aerial tissues than in subterranean organs. The GUS histochemical staining analysis revealed that the gene exhibits a tissue-specific inducible expression pattern under drought stress conditions, with its promoter activity being specifically activated in the leaf and stem base regions, while no detectable activity was observed under normal growth conditions (Supplementary Figure S7C,D). These findings indicate that *OsNAC25* primarily functions through aerial tissue-specific expression induced by drought stress. This contrasts with previously reported NACs from the SNAC subgroup that predominantly exhibit stronger root-specific than leaf-specific expression patterns when responding to abiotic stresses [27,28,29]. Therefore, unlike SNACs, *OsNAC25* principally operates as a stress-inducible transcription factor in aboveground plant tissues.

The overexpression of *OsNAC25* enhances drought stress tolerance in transgenic materials, accompanied by elevated activities of peroxidase (POD) and catalase (CAT), reduced levels of malondialdehyde (MDA)—a harmful oxidative damage marker— and decreased superoxide dismutase (SOD) activity. Plants have evolved a sophisticated antioxidant system comprising both non-enzymatic and enzymatic components [30,31]. While sustained high levels of antioxidant enzymes (POD, CAT, SOD) are generally associated with ROS scavenging and stress tolerance, we observed a paradoxical reduction in SOD activity in overexpression lines. This suggests that the oxidative process involves more than just the connection between a single organelle and the nucleus. The cellular redox signaling hub may be seen as a key integrator of retrograde signals arising from different organelles, allowing communication between different cellular compartments and the nucleus [32]. This divergence suggests that *OsNAC25* may not participate in SOD-mediated regulatory pathways.

RNA-seq analysis of *OsNAC25* material: The transcriptomic profiling of *OsNAC25* focused on cellular/metabolic processes, biological regulation, response to stimuli, cellular anatomical entities, intracellular organelle functions, molecular binding, and catalytic activity (Figure 4E,F). The most significant pathways associated with cellular/metabolic processes were phenylpropanoid biosynthesis and glutathione metabolism. The phenylpropanoid biosynthesis pathway was predominantly enriched with peroxidase- and β-glucosidase-related genes. Plants possess antioxidant systems to mitigate oxidative damage, and these pathways directly correlate with enhanced antioxidant capacity, playing a pivotal role in improving drought tolerance [33]. Glutathione is critically involved in drought stress responses and ROS scavenging [34]. Under drought conditions, rice crops exhibit a well-documented reduction in glutathione levels, followed by a significant compensatory increase as an adaptive response [31]. Multiple studies have proposed glutathione depletion as a potential biomarker for screening drought-resistant plant varieties [34,35]. Regarding biological regulation and stress responses, phytohormone signaling transduction—particularly pathways involving IAA (indole-3-acetic acid) and ABA (abscisic acid)—emerged as a crucial mechanism influencing plant growth and stress adaptation. Our KEGG enrichment analysis revealed substantial modifications in multiple phytohormone biosynthesis pathways.

Numerous binding pathways and catalytic activity-related processes were significantly influenced by *OsNAC25*. Notably, *OsNAC25* modulated diterpenoid biosynthesis through pronounced effects on carbon metabolism and GA44-dioxygenase synthesis, establishing a direct linkage to drought stress adaptation [36]. Photosynthesis, the primary driver of plant growth, provides essential energy for organic compound synthesis [36,37]. Extensive crop improvement research has prioritized the identification of quantitative trait loci (QTLs) associated with photosynthetic enhancement [38]. Ferredoxin-encoding genes, integral to photosynthetic electron transport, maintain intracellular redox homeostasis. Intracellular organelles coordinate a complex network of thousands of genes governing metabolic, signaling, and biosynthetic functions [39]. Hierarchical clustering analysis further elucidated drought-responsive molecular mechanisms, revealing *OsNAC25*’s systemic regulatory role across these biological hierarchies.

Collectively, our integrated transcriptomic and physiological analyses established *OsNAC25* as a nucleo-cytoplasmic transcription factor that regulates drought resilience in rice. *OsNAC25* enhances drought tolerance by reducing oxidative damage, promoting ROS scavenging, modulating hormone signaling, and activating antioxidant-related pathways. This discovery offers a promising target for the molecular breeding of drought-resistant crops.

## 4. Materials and Methods

### 4.1. Plant Materials and Growth Conditions

Wild-type rice (*Oryza sativa* cv. Zhonghua 11, ZH11) seeds were used in this study. All rice lines were propagated in paddy fields at Hunan Agricultural University. Rice seedlings were cultivated in a greenhouse under a 16-hour light/8-hour dark photoperiod at 30 °C. Transgenic overexpression lines were constructed using the *OsNAC25* (LOC_Os11g31330) coding sequence (CDS) cloned into a modified T-DNA vector under the control of the ubiquitin promoter, with a 3 × FLAG tag fused to its C-terminus. *OsNAC25* knockout mutants were generated via the pYLCRISPR/Cas9Pubi-H system. All plants were screened for homozygous lines at the T2 generation.

### 4.2. Subcellular Localization of OsNAC25 in Rice Protoplasts

To investigate the subcellular localization of *OsNAC25*, the primers mCherry-*OsNAC25*-F and mCherry-*OsNAC25*-R were designed using Vector NTI. The full-length coding sequence (CDS) of *OsNAC25* was amplified from wild-type ZH11 cDNA and cloned into a fusion expression vector containing the mCherry fluorescent tag. The resulting mCherry-*OsNAC25* construct was co-transfected with the RPL-CFP nuclear localization marker into rice protoplasts via PEG4000-mediated transformation. Transfected protoplasts were incubated in darkness at 28 °C for 16 h, and fluorescence signals were visualized using a Zeiss LSM780 confocal laser microscope (Carl Zeiss, Jena, Germany).

### 4.3. RNA Extraction and Quantitative Real-Time RT-PCR (qRT-PCR) Analysis

Total RNA was extracted from rice seedling leaves using the TransZol Up Plus RNA Kit (TransGen Biotech, Beijing, China). First-strand cDNA synthesis was performed with ≥1 μg of total RNA using the HiScript First Strand cDNA Synthesis Kit (Vazyme, Nanjing, China). Gene expression levels were analyzed by qRT-PCR with Hieff qPCR SYBR Green Master Mix (YEASEN, Shanghai, China). Data were normalized to the reference gene Actin1 and quantified using the relative quantitative method (2−ΔΔCT method). Three biological replicates were conducted for each experiment. The primers used for qRT-PCR in this study are listed in Table S2.

### 4.4. Enzyme Activity Assays

To determine the activities of MDA (malondialdehyde), CAT (catalase), SOD (superoxide dismutase), and POD (peroxidase), the corresponding assay kits (Solarbio Science & Technology Co., Ltd., Beijing, China) were employed according to the manufacturers’ protocols. Fresh rice seedling leaf samples were collected and immediately frozen in liquid nitrogen. For analysis, 0.1 g of tissue was homogenized in 1 mL of ice-cold extraction buffer. The homogenate was centrifuged at 8000× *g* for 10 min at 4 °C, and the supernatant was collected for enzymatic activity assays.

### 4.5. Transcriptome Analysis

To investigate the functional role of *OsNAC25* in drought stress, RNA sequencing (RNA-seq) was conducted using wild-type (ZH11), *OsNAC25*-cr (CRISPR/Cas9 knockout mutants), and *OsNAC25*-OE (overexpression) lines. The experiment used 10-day-old seedlings grown under normal conditions as the controls. The treatment groups were grown under identical standard conditions for 9 days until reaching the two-leaf-one-heart stage, and then subjected to 24-hour drought stress treatment. For RNA extraction, a pooled sampling approach was adopted: each sample (e.g., *OsNAC25*-cr under control conditions) comprised a homogenate of tissues from eight individual plants, representing one biological replicate. Three biological replicates were established per treatment group, resulting in six experimental groups (ZH11, *OsNAC25*-cr, and *OsNAC25*-OE, each with control and drought-treated subgroups) and 18 total samples. Sequencing was performed by Baimaike Biotechnology (Beijing, China). The raw data were quality assessed using FastQC, followed by adapter and low-quality read removal (Q-score < 20) with Trimmomatic. Differential expression analysis was performed using the DESeqR package (1.10.1) with stringent thresholds (|log2(fold change)| > 1 and adjusted *p*-value < 0.01). The gene ontology (GO) enrichment analysis of differentially expressed genes was conducted using the clusterProfiler R (4.4.4) package, with KEGG pathway enrichment analysis also performed using the same package, using the whole-genome background as reference for statistical significance (adjusted *p*-value < 0.05). To validate RNA-seq reliability, eight randomly selected genes were analyzed via qRT-PCR using Actin as the internal reference gene. Relative expression levels were quantified before and after drought treatment. All assays included three biological replicates under rigorously standardized conditions. 

## Figures and Tables

**Table 1:** Key candidate genes under drought conditions (ZH11 and knockout mutants as controls versus overexpression lines).

Gene Symbols	gene_name	ZH11vsOE (FDR = 0.01, FC = 2)	cr vs OE (FDR = 0.01, FC = 2)
*OsPrx30*	Os02g0240100	down	down
*Os3BGlu6*	Os03g0212800	down	down
*OsGAD2*	Os04g0447800	down	down
*OsIAA14*	Os03g0797800	down	down
*OsPIL16*	Os05g0139100	down	down
*OsPYL4*	Os03g0297600	down	down
*OsCatC*	Os03g0131200	up	up
*OsKSL12*	Os02g0568700	down	down
*OsPPDKA*	Os03g0432100	up	up
*OsFdC1*	Os03g0659200	down	down

## Data Availability

The raw RNA-seq data reported in this paper have been deposited in the Genome Sequence Archive in National Genomics Data Center, China National Center for Bioinformation/Beijing Institute of Genomics, Chinese Academy of Sciences (GSA: CRA025850) that are publicly accessible at https://ngdc.cncb.ac.cn/gsa (accessed on 14 May 2025).

## Supplementary Materials

The following supporting information can be downloaded at: https://www.mdpi.com/article/10.3390/ijms26104954/s1.
