# Supplementary material for: The OsNAC25 Transcription Factor Enhances Drought Tolerance in Rice
Source: Int J Mol Sci. 2025 May 21;26(10):4954. doi: 10.3390/ijms26104954 (PMC12112375; doi:10.3390/ijms26104954)
Supplement: Supplementary file 1 [file ijms-26-04954-s001.zip › Supplementary Figures.pdf]

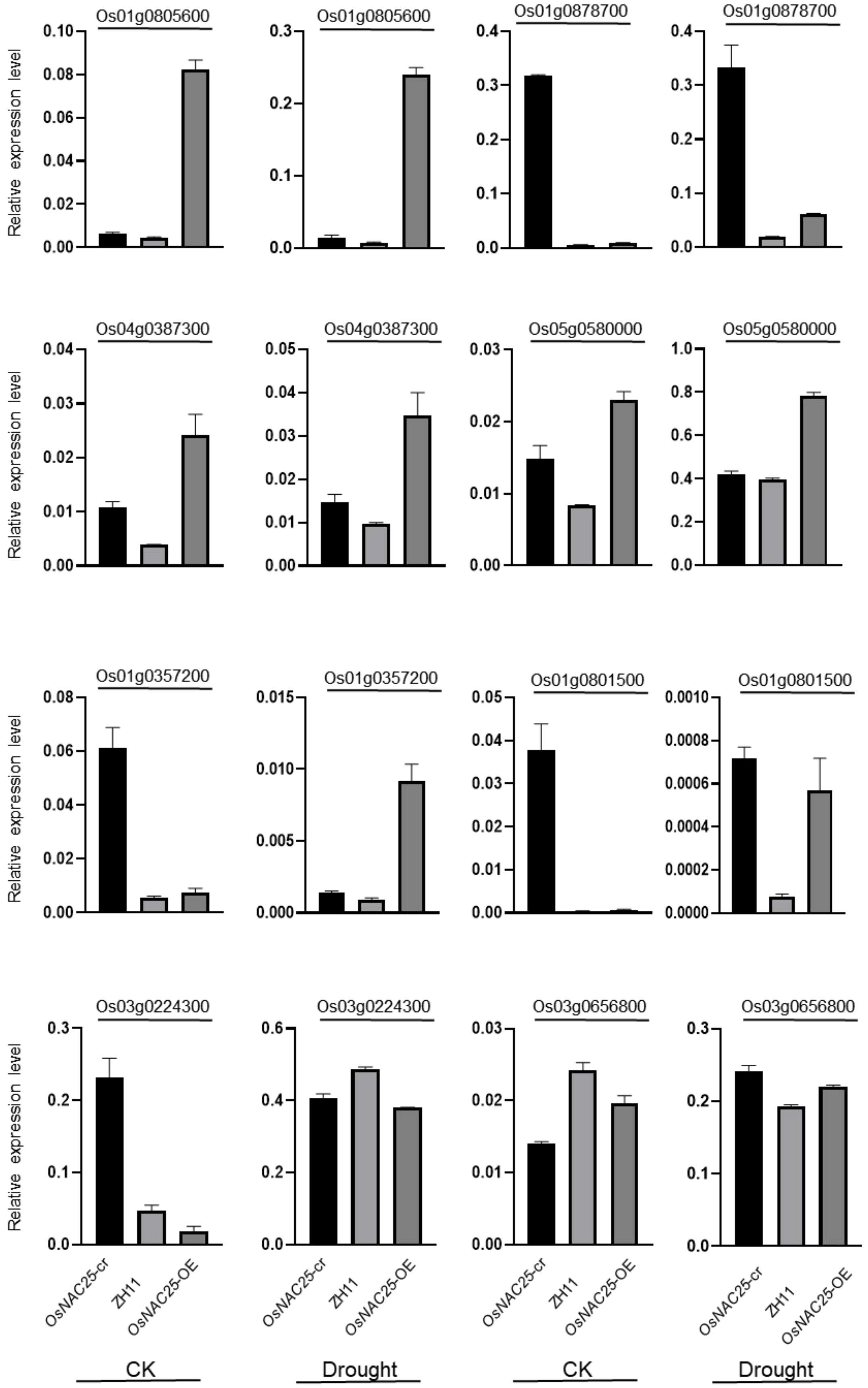

Supplementary Figure S1. Validation of the RNA-seq results by qRT-PCR. Error bars indicate the se based on three technical replicates.

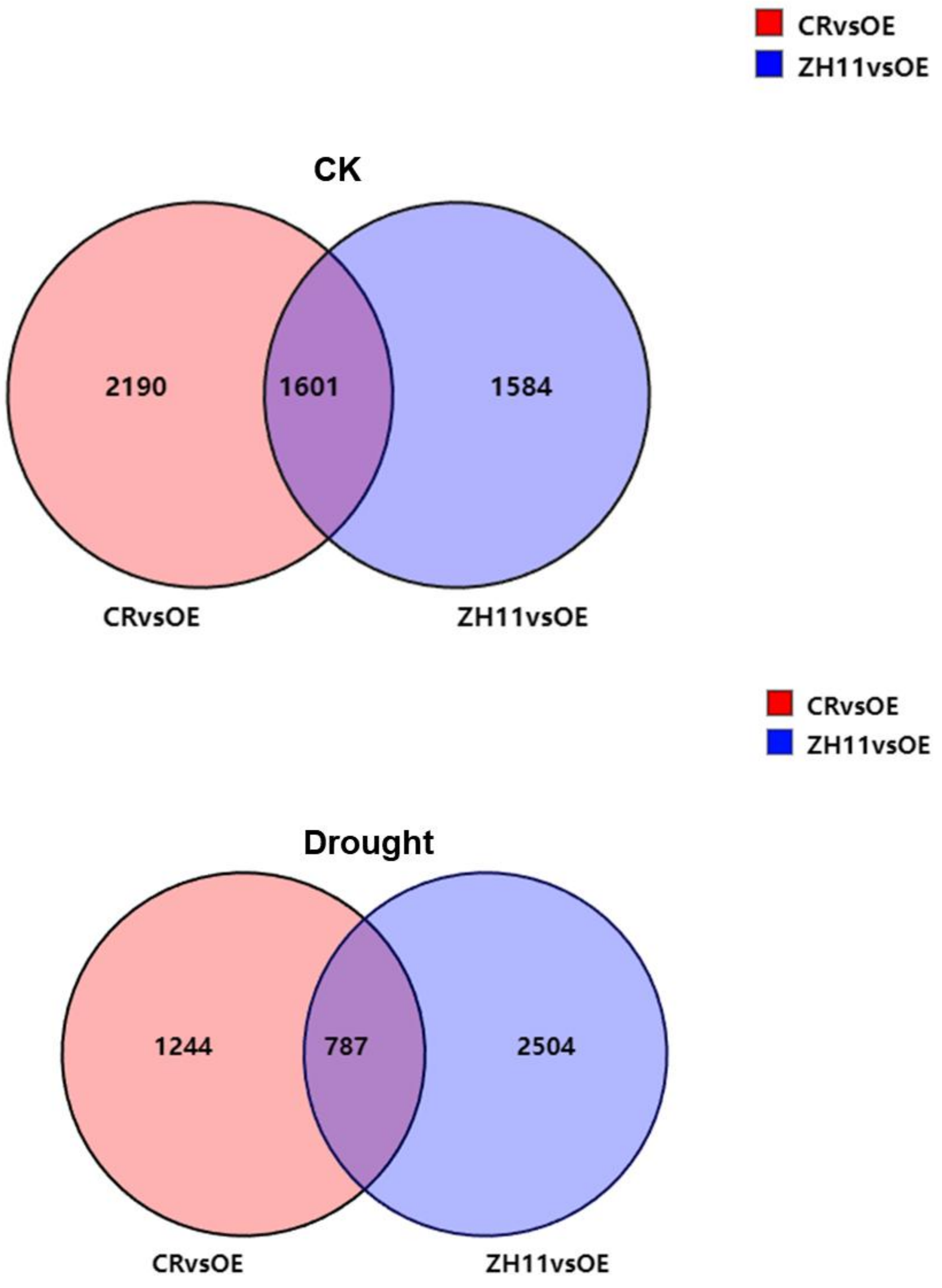

Supplementary Figure S2. Venn diagram analysis of the DEGs.

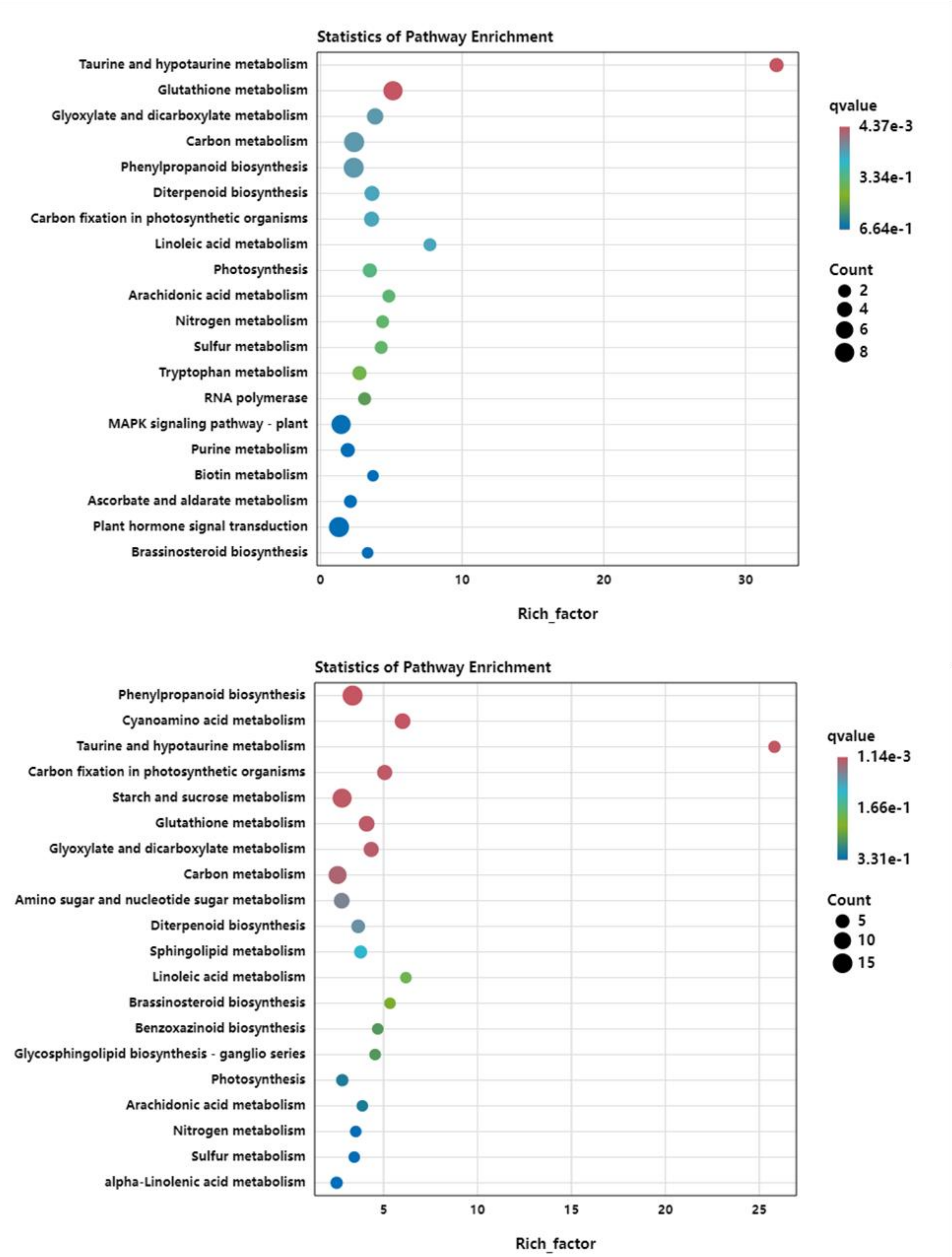

Supplementary Figure S3. KEGG analysis of cellular and metabolic process DEGs.

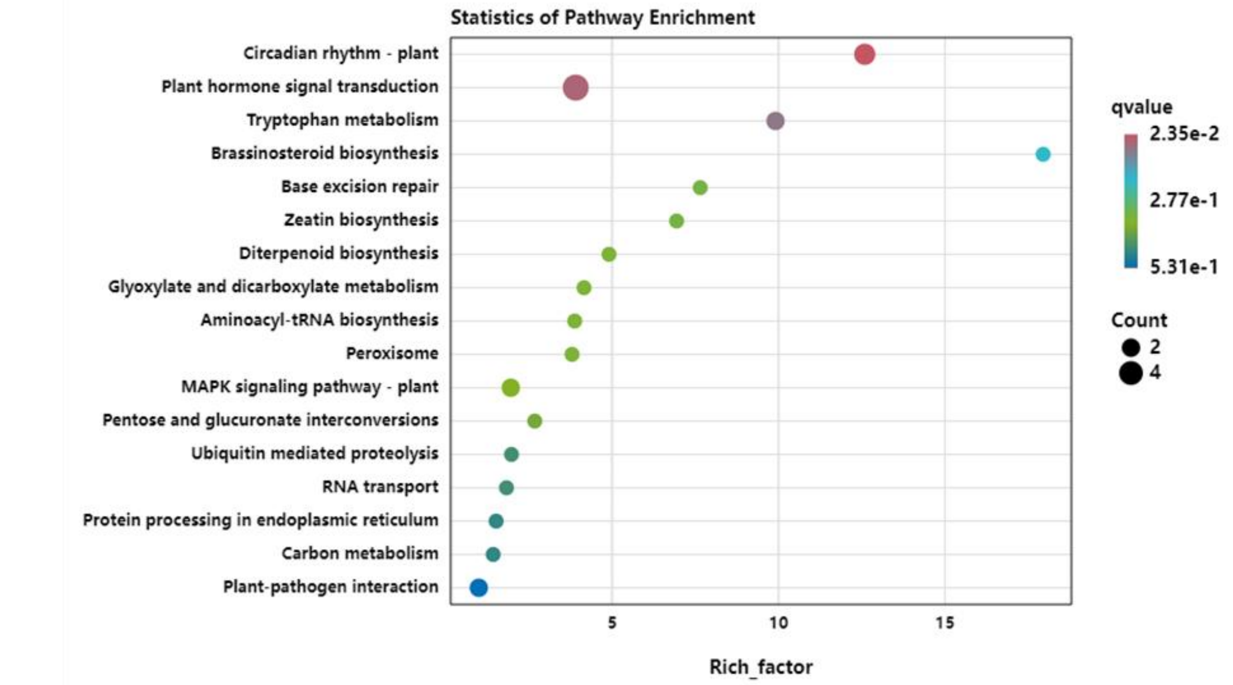

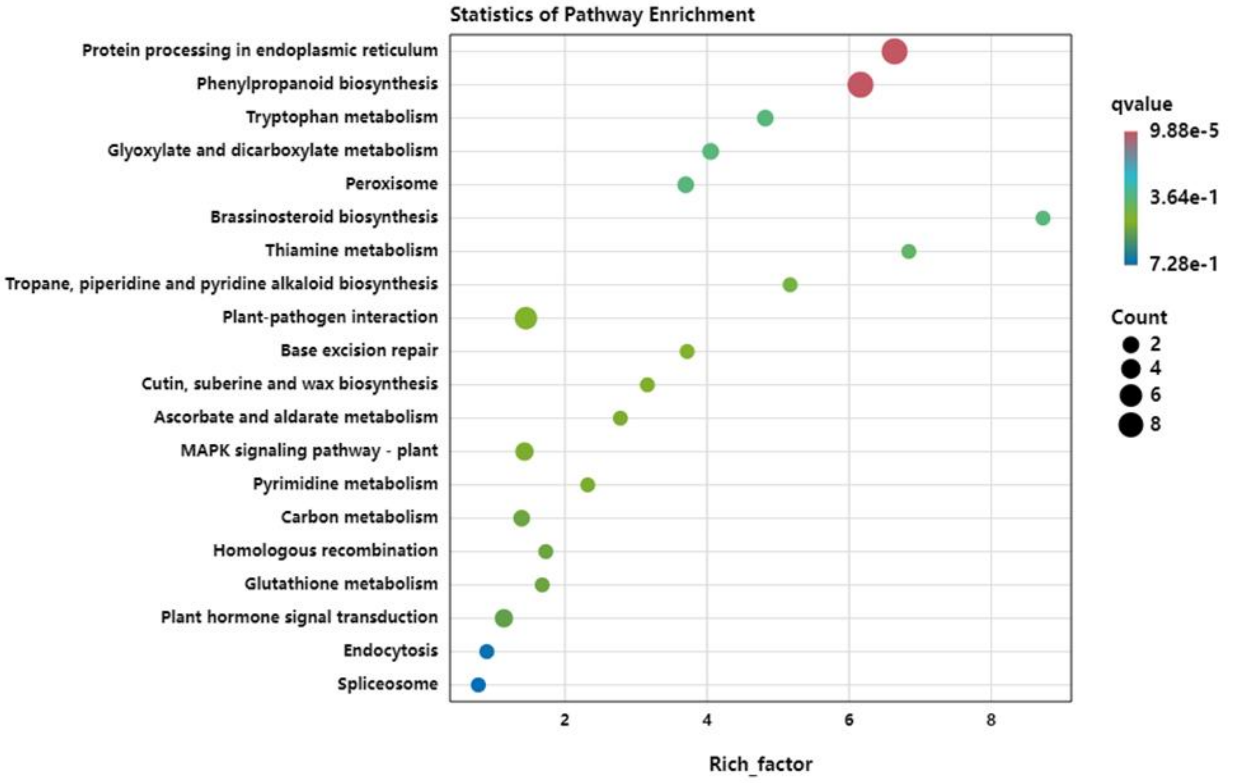

Supplementary Figure S4. KEGG analysis of biological regulation and response to stimulus DEGs.

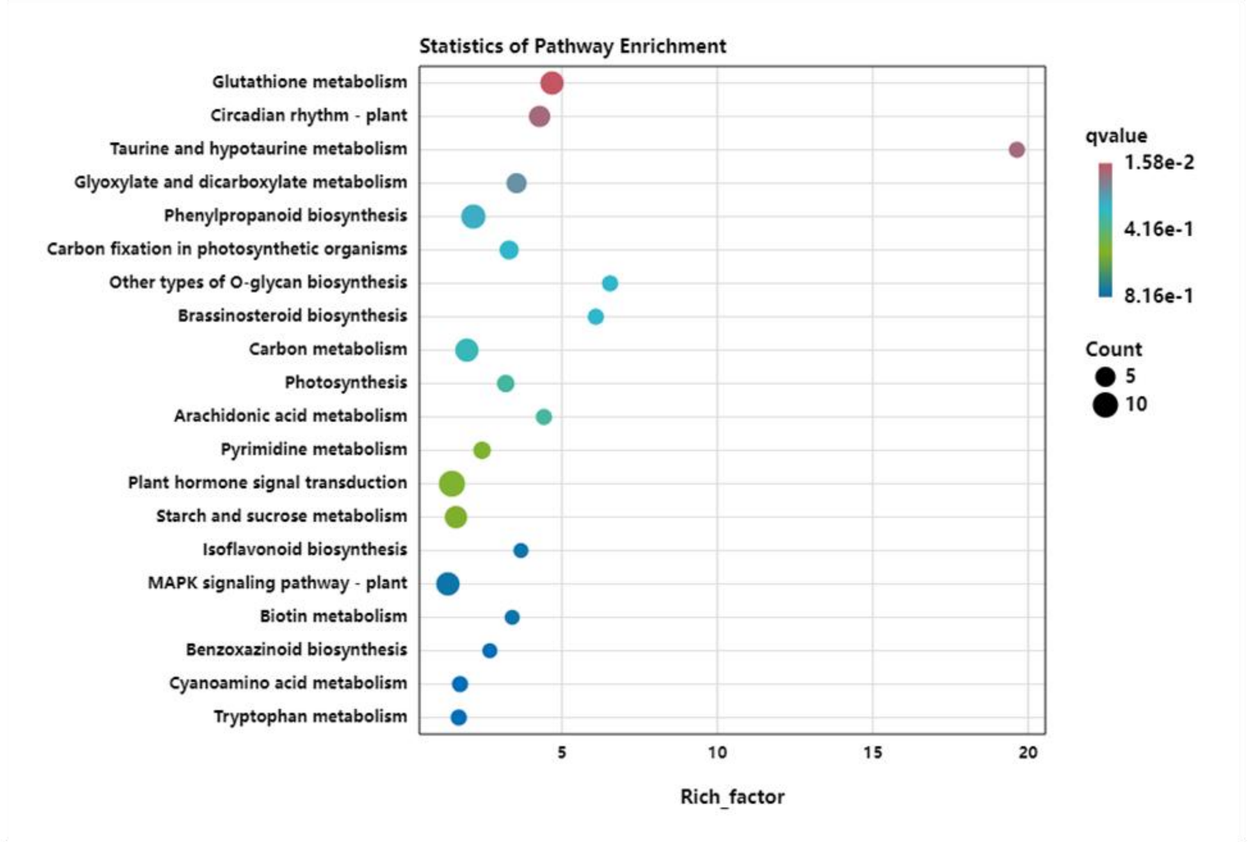

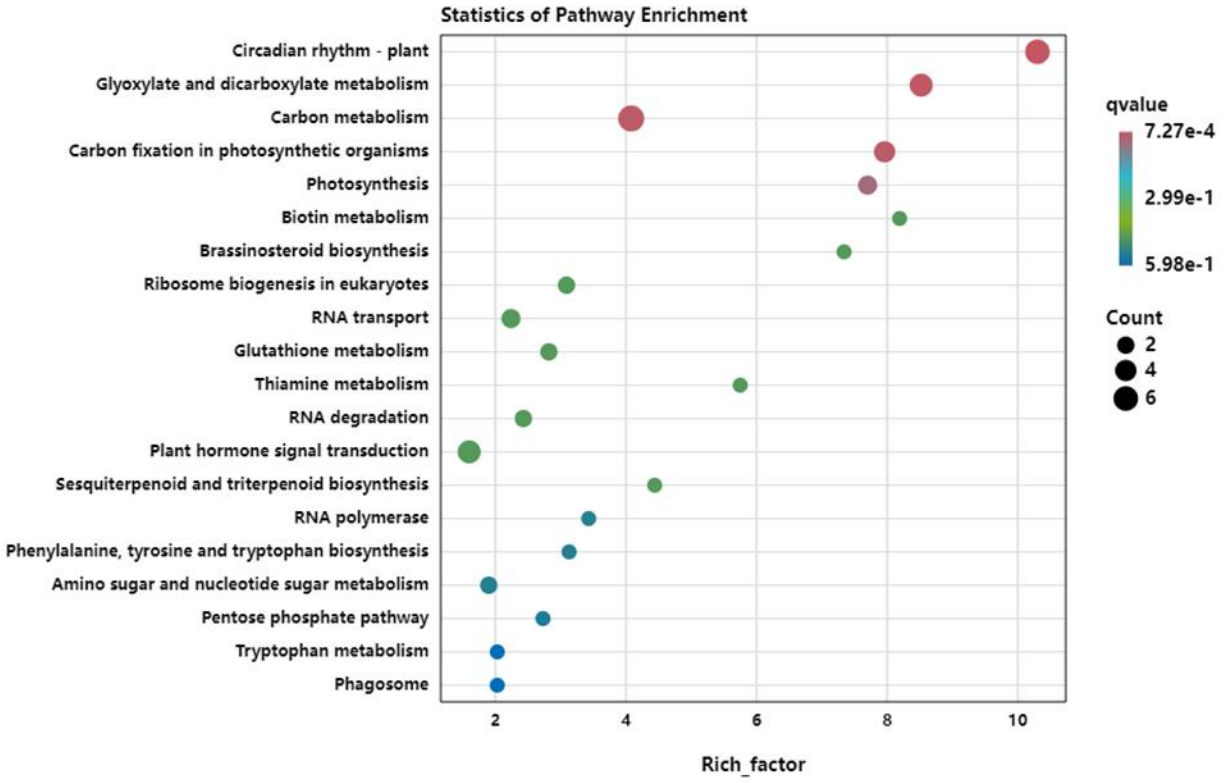

Supplementary Figure S5. KEGG analysis of cellular anatomical entity and intracellular DEGs.

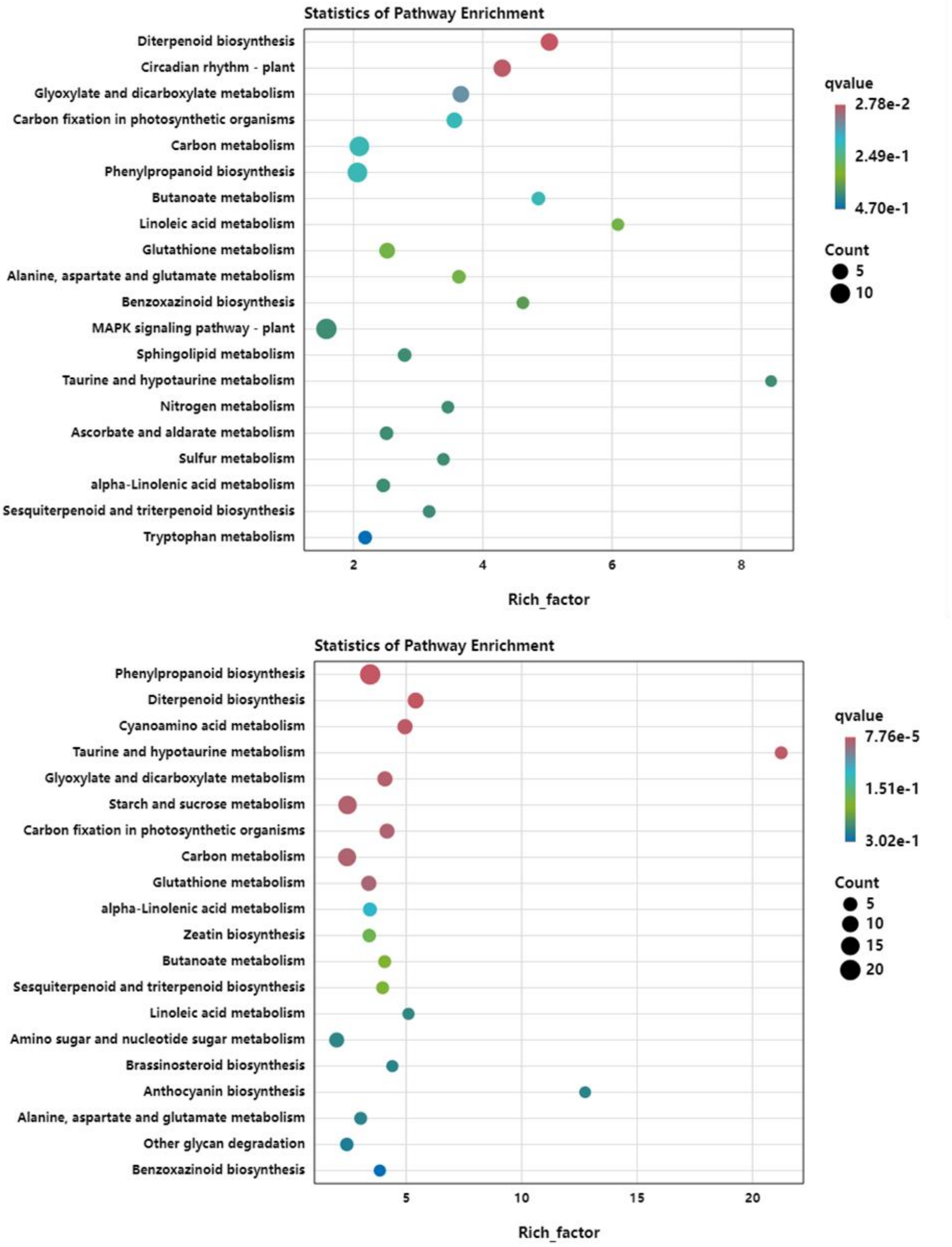

Supplementary Figure S6. KEGG analysis of binding and catalytic activity DEGs.

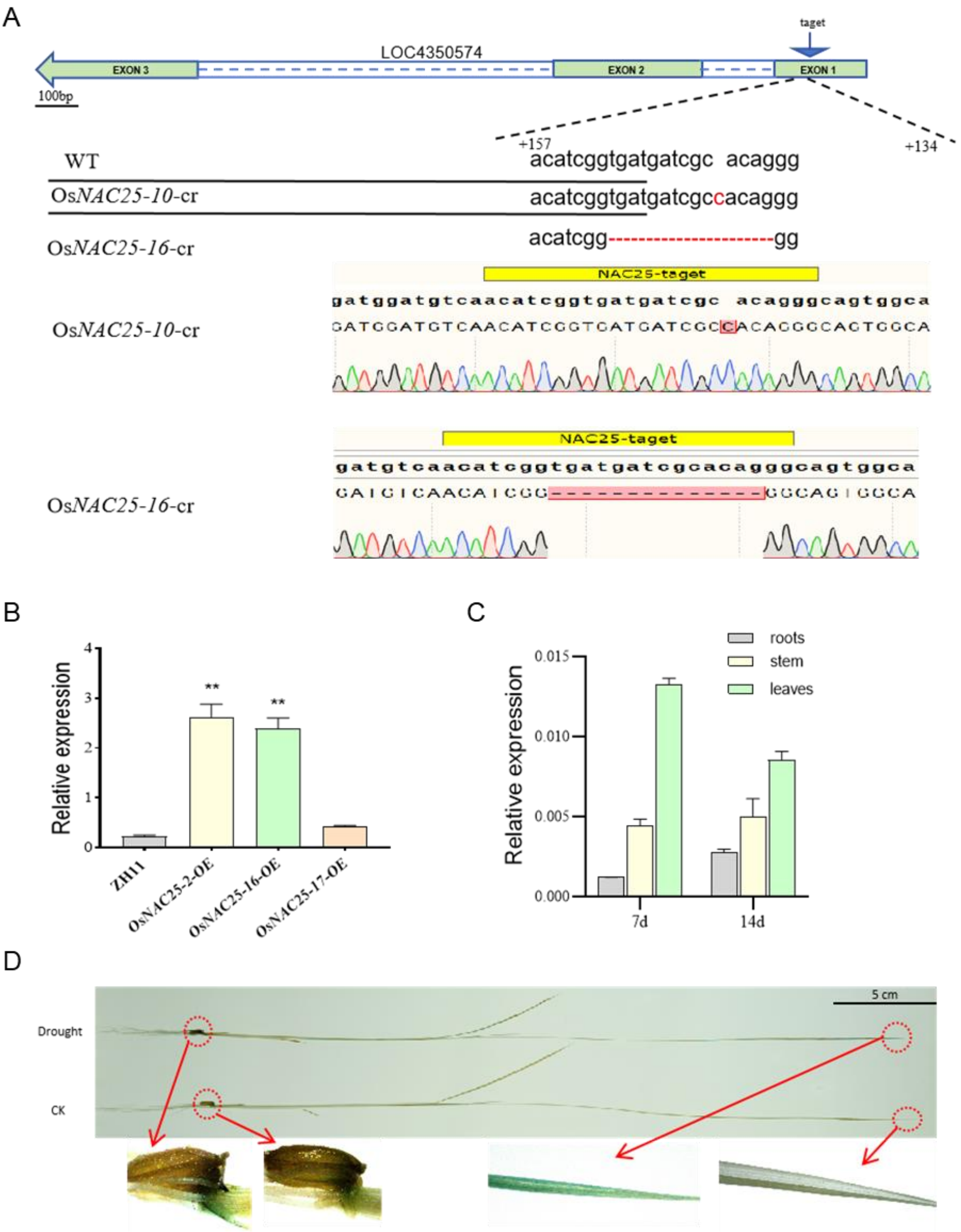

Supplementary Figure S7. *OsNAC25* knock-out mutants and Overexpression of a *OsNAC25* transcription factor. (A) Schematic illustration of the target sites in the *OsNAC25* genomic sequence. Mutation sequences are in red. (B) The expression levels of the *OsNAC25* transcription factor in overexpression materials. (C) Relative expression levels in roots, stems, and leaves of rice (Oryza sativa) at 1-week and 2-week leaf ages. (D) GUS staining of *OsNAC25* materials. The drought treatment (Drought) involved 9 days of hydroponic culture followed by 1 day of drought stress, while the control (CK) was maintained in hydroponic culture for 10 days. The insets below are magnified 10-fold.
