# Supplementary material for: The OsNAC25 Transcription Factor Enhances Drought Tolerance in Rice
Source: Int J Mol Sci. 2025 May 21;26(10):4954. doi: 10.3390/ijms26104954 (PMC12112375; doi:10.3390/ijms26104954)
Supplement: Supplementary file 1 [file ijms-26-04954-s001.zip › Supplementary Tables.pdf]

Supplementary Table S1. Results of sequencing data quality control and comparison analysis.

Sample name	Clean reads	Clean bases	GC content(%)	⩾Q30 (%)
ZH11-CK1	19 823 346	5 941 642 287	51.18	96.45
ZH11-CK2	20 309 648	6 083 692 778	51.23	94.96
ZH11-CK3	20 354 212	6 100 729 243	50.64	93.57
OsNAC25-OE-CK1	21 861 334	6 548 239 871	50.76	97.73
OsNAC25-OE-CK2	20 122 091	6 031 306 068	50.40	97.04
OsNAC25-OE-CK3	19 616 942	5 879 375 315	49.96	94.51
OsNAC25-cr-CK1	20 006 010	5 996 995 608	50.93	96.61
OsNAC25-cr-CK2	20 827 120	6 240 483 416	51.37	97.58
OsNAC25-cr-CK3	19 837 371	5 946 705 550	51.07	97.16
ZH11-D1	19,688,056	5,891,285,774	51.43	96.28
ZH11-D2	19,194,554	5,750,246,661	51.58	95.58
ZH11-D3	20,160,442	6,027,857,086	52.44	96.38
OsNAC25-OE-D1	26,587,909	7,965,754,149	51.62	96.96
OsNAC25-OE-D2	19,455,829	5,829,380,205	51.56	93.07
OsNAC25-OE-D3	20,463,345	6,131,338,356	51.59	93.35
OsNAC25-cr-D1	20,550,100	6,155,655,374	51.39	93.75
OsNAC25-cr-D2	20,207,216	6,053,268,747	51.28	93.79
OsNAC25-cr-D3	19,716,045	5,905,790,485	51.32	94.87

Supplementary Table S2. Oligonucleotides used in this study.

Primer	Primer sequence
OsActin-RT-F	TTGGCTCCTAGCAGCATGAA
OsActin-RT-R	CCTTGGCAATCCACATCTGC
OsNAC25-RT-F	TCGTCCCAGTGGCGGAGAG
OsNAC25-RT-R	TGGGCACCTCCGAGCCTG
Os01g0805600- RT-F	CCGCCTACCGGAAGGCAA
Os01g0805600- RT-R	GGCTTGACAGGCGCCTTC
Os01g0878700- RT-F	ATGGTGGGTTACACCATCAC

Os01g0878700- RT-R	ACGGAGATGAGCGTCACC
Os04g0387300- RT-F	GGGCAAGAAAGGTGGTGG
Os04g0387300- RT-R	TCCACAGCCTCCCCTCCT
Os05g0580000- RT-F	CGATATCCCCATGAGCAACT
Os05g0580000- RT-R	AGGCATTTGTGTAGCGGC
Os01g0357200- RT-F	TGTCCACCTCGCGTCCGG
Os01g0357200- RT-R	AATCGTCGTCCGTGAAGG
Os01g0801500- RT-F	CCAGCAACATCACCGACA
Os01g0801500- RT-R	TACGACTGGACCCACGACG
Os03g0224300- RT-F	AGTCGTACAACTGCGTGC
Os03g0224300- RT-R	GTTCGCCGACACGTACGA
Os03g0656800- RT-F	GGCACGTTCCTGAACGTG
Os03g0656800- RT-R	TCATCAGCCCGGCGATGT
